# Perceived Utility of Jan Aushadhi Outlets and Awareness of Generic Medicines at the Andaman and Nicobar Islands: A Cross-Sectional Study

**DOI:** 10.7759/cureus.57630

**Published:** 2024-04-04

**Authors:** Akshat Chaturvedi, Ashok K Dubey, Avula Naveen, Marpu Raghava Sravani

**Affiliations:** 1 Internal Medicine, Andaman and Nicobar Islands Institute of Medical Sciences Port Blair, Port Blair, IND; 2 Pharmacology, All India Institute of Medical Sciences (AIIMS) Bilaspur, Bilaspur, IND; 3 Pharmacology and Therapeutics, All India Institute of Medical Sciences (AIIMS) Bilaspur, Bilaspur, IND; 4 Pharmacology and Therapeutics, Mamata Medical College, Khammam, IND

**Keywords:** public health, knowledge, generic medicine, jan aushadhi kendra, jan aushadhi scheme

## Abstract

Background: Despite being the leading exporter of generic medicines to the world, affordable medicines are still beyond the reach of most patients in India. Analysis of the National Sample Survey data showed that in one year, more than 55 million Indians became poor only because they had to spend their own money to purchase medicines. The Jan Aushadhi Scheme launched by the Government of India is an ambitious step to make quality generic drugs affordable to the common man of the country.

Objective: This study aimed to assess the knowledge, attitude, and perceptions of patients at a tertiary care teaching hospital in the Andaman and Nicobar Islands about Jan Aushadhi Kendras and generic medicines.

Materials and methods: The study was a questionnaire-based cross-sectional study. A prevalidated self-made questionnaire was distributed to 200 patients visiting the OPD of different clinical departments in the hospital. Participants' knowledge, attitude, and perception of Jan Aushadhi Kendras and generic medicines were evaluated. Analysis of collected data was done using descriptive statistical measures such as mean and percentages.

Results: It was found that most of the participants were not fully aware of the Jan Aushadhi Scheme and the facts about generic medicines. The majority of the participants were under the notion that generics were not similar in quality to the branded ones.

Conclusion: The study observed that the patients had a very poor understanding of the Jan Aushadhi Scheme and generic medicines with the majority being ignorant and having incorrect information. To fill this gap, a more proactive approach by the healthcare workers and authorities is needed to disseminate the scheme-related facts, dispel the myths regarding generics, and accept the program wholeheartedly by the common man.

## Introduction

Despite being the world's leading exporter of generic medicines, affordable medicines are still beyond the reach of most of the patients in India [1]. According to the National Health Accounts Estimates, the out-of-pocket expenditure in India accounts for 52% of health expenditure for the year 2019-2020, and it is one of the highest in the world. A cross-sectional study examining various National Statistical Office (NSO) surveys reported that 55 million people in India were pushed into poverty during the 1994-2014 period due to out-of-pocket health expenditure [2,3]. One of the ways to decrease this enormous economic burden incurred due to expenditure on medications is the use of good-quality but cheaper generic drugs that are as effective as their branded versions [4].

Prescribing generic drugs instead of branded versions is being promoted nowadays and has become an accepted practice in many parts of the world, but it has not yet gained a popularly accepted status in India. This could be attributed to various factors, including the non-availability of generic drugs, lack of trust among physicians for generic medicines due to perceived doubts related to the quality of the drugs, and lack of proper awareness about these formulations in the general population [5]. To relieve the common people from the financial burden caused by expensive branded medicines, the Government of India has launched Pradhan Mantri Bharatiya Janaushadhi Pariyojna, also known as Jan Aushadhi Yojna. This program aims to provide quality generic medicines at affordable prices to the masses through special outlets known as Jan Aushadhi Kendras, as against the costly branded drugs commonly available at most chemist shops [6].

The availability of scientific data on the experience and attitude of consumers of generic medicines through Jan Aushadhi outlets is crucial for better policy implementation, but there is scarce insight due to very few studies on this topic. Studies in similar areas have been conducted in some other countries, where retail pharmacies make generic drugs available, but that scenario is unlike the government-initiated launch of outlets in India [7-12]. There haven't been sufficient studies to provide robust feedback on the knowledge and attitude of the common man about the policy since it was launched.

The Andaman and Nicobar Islands (ANIs), an Indian Union Territory, is an archipelago of more than 500 islands situated in the Bay of Bengal, about 1,200 km away from the mainland of peninsular India [13]. The Directorate of Health Services, Andaman and Nicobar administration set up the first such Jan Aushadhi Kendra of the state on the premises of G. B. Pant Hospital, Port Blair, on October 10, 2018. This study was planned to assess the perception of the patients about the Jan Aushadhi outlets and the gaps, if any, in the intent and the successful outreach of the program in the remote ANIs. Understanding the impact of this initiative and finding out the perceptive or logistic challenges, if any, through this study may help in suggesting measures to reinforce the importance of Jan Aushadhi Kendras and generic medicines. To the best of our knowledge, no study has been done earlier, regarding the generic drug dispensing government outlets for common people.

## Materials and methods

This was a descriptive cross-sectional study. It was conducted at the only tertiary care teaching hospital located in ANIs, India, catering to the entire islands. The study was initiated after obtaining approval from the Institutional Ethics Committee of the Andaman and Nicobar Islands Institute of Medical Sciences (ANIIMS) Port Blair (approval number: ANIIMS/IEC/2022-23/28). The study was conducted at G. B. Pant Hospital in February and March 2023. Before the initiation of the study, participants were detailed about the project and its significance in providing affordable healthcare and promotion. We also assured the patients about the anonymity of their participation in the study. A pre-validated, self-made questionnaire was distributed to the study participants who gave their consent to participate in the study. A sample size of convenience was decided as per the duration and feasibility. A total of 200 patients visiting the hospital's outpatient department participated in the study. Patients aged above 18 years and who had given consent to participate in the study were included. In contrast, patients with severe ailments were excluded from the study.

The questionnaire was divided into different sections with information about the demographic profile of participants, their knowledge about generic medicines, and the perceived utility of Jan Aushadhi Kendras. There were 16 items in the questionnaire, and participant's knowledge, attitude, and perception were assessed with yes or no options. We collected the questionnaire forms from the patients and evaluated the same for their completeness and legibility. Then we entered the pooled data in an Excel sheet for further analysis. Appropriate statistical measures such as means and percentages were used for tabulating the results.

## Results

Out of 200 participants, 116 (58%) were males, and the remaining 84 (42%) were females (Table 1). Demographic data revealed that the majority of the patients, 62 (31%), belonged to the 41-50-year age category, 51 (25.5%) belonged to 51-60 years, 33 (16.5%) belonged to 31-40 years, 32 (16%) belonged to above 60 years, and lastly 22 (11%) belonged to the 18-30-year category (Table 2). Knowledge, attitude, and perception of patients about Jan Aushadhi Kendras and generic medicines are explained in Table 3.

**Table 1:** Gender-wise distribution of participants

S. no.	Gender-wise distribution of the participants	Number	Percentage
1	Male	116	58
2	Female	84	42

**Table 2:** Age-wise distribution of the participants

S. no.	Age-wise distribution of the participants	Number	Percentage
1	18-30 years	22	11
2	31-40 years	33	16.5
3	41-50 years	62	31
4	51-60 years	51	25.5
5	Above 61 years	32	16

**Table 3:** Knowledge, attitude, and perception of patients about Jan Aushadhi Kendras and generic medicines

S. no.	Variable (question)	Yes number, %	No number, %
1	Are you aware of the scheme called "Pradhan Mantri Bharatiya Jan Aushadhi Pariyojna"?	64 (32%)	136 (68%)
2	Are you aware about Jan Aushadhi Kendra?	90 (45%)	110 (55%)
3	Have you ever visited any Jan Aushadhi Kendra?	72 (36%)	128 (64%)
4	Have you ever heard about generic medicines?	124 (62%)	76 (38%)
5	Have you ever purchased generic medicines?	65 (32.5%)	135 (67.5%)
6	Do you think generic drugs are cheaper than branded drugs?	58 (29%)	142 (71%)
7	Do you know the difference between branded and generic medicines?	24 (12%)	176 (88%)
8	Have you ever requested a pharmacist to dispense you with generic medicines?	12 (6%)	188 (94%)
9	Are you aware of government regulations on generic and branded medicines?	72 (36%)	128 (64%)
10	Did you find the medicines available at Jan Aushadhi Kendras equally effective?	58 (29%)	142 (71%)
11	Have you ever requested your doctor to prescribe generic medicines?	12 (6%)	188 (94%)
12	Do you think generic drugs are safe?	58 (29%)	142 (71%)
13	Do you know that generic medicines are as effective as branded medicines?	58 (29%)	142 (71%)
14	Do you think the government should come up with stricter laws for the implementation of the Jan Aushadhi Medicine Scheme?	184 (92%)	16 (8%)
15	Do you think there is a need for training programs for healthcare providers to increase awareness regarding generic medicines?	152 (76%)	48 (24%)
16	Would you like to know more about the Jan Aushadhi Scheme through media, newspapers, etc.?	200 (100%)	0

It was found that many of the participants were not fully aware of the Jan Aushadhi Scheme. Less than half (45%) of the participants knew about Jan Aushadhi Kendras, whereas 58% were clueless about the type of drugs that they were getting at the Jan Aushadhi Kendras. Some of the patients (10%) believed that such centers provide branded medicines, while only 26% knew that these outlets provide generic drugs, and the remaining 6% believed that both generic and branded medicines are sold there.

Among the participants, 58 (29%) believed that generic medicines were less costly but still of the quality comparable to the branded alternatives, but the majority of the participants were under the notion that generics are not similar to the branded ones.

The existence of any such government scheme as the Jan Aushadhi program, which was making quality generic drugs available at cheaper prices, was only known to 64 (32%) of the participants. This relative lack of awareness was similar across participants of all ages.

When a question was asked about the quality of the medicines, 38% of the participants didn't know about the difference between generic and branded medicines; 16% considered branded medicines to be superior, and 9% were not sure as they considered generic drugs to be superior in efficacy. The remaining 37% considered both generic and branded drugs to be equally good.

A major proportion (64%) of participants under study did not know of the regulations and guidelines governing generic and branded drugs. About 40% of participants had no idea of the choices about the types of drugs to be purchased, and 32% of participants were of the opinion that both generic and branded medicines should be procured. Out of the rest, 16% thought that they should buy generic drugs, and 12% were in favor of buying branded medicines.

A total of 152 (76%) participants believed that healthcare providers should be trained to increase awareness regarding Jan Aushadhi outlets and the drugs being dispensed through the same. Almost everyone responded positively to receiving further information on generics and the Jan Aushadhi Scheme.

## Discussion

The current study revealed that most of the patients were neither aware of the Jan Aushadhi Scheme nor of the existence of government outlets for distributing generic drugs. Less than one-third of the patients knew that generic medicines are cheaper and safe and they wanted Jan Aushadhi Kendras to be opened at every gram panchayat level so that the healthcare burden on poor families would be reduced. In this study, only 29% of the patients were aware about Jan Aushadhi medicines being comparable in efficacy to brand medicines despite being cheaper. This was mainly due to a lack of awareness among the patients about the Jan Aushadhi medicines and the program. 

When we educated them about the concept of the Jan Aushadhi Scheme and generic medicines, almost all patients opined that more such outlets should be opened in different parts of the city and the information regarding those should be disseminated through social media platforms and local newspapers. Most of the patients also believed that the policy could be implemented more effectively if the government came up with stricter guidelines related to the prescribing of generic drugs.

Similar to the current study, an earlier study by Skaltsas and Vasileiou conducted in Greece found that only 33.1% of the patients knew that the generic drugs were not different from the branded drugs in composition, while only 26.3% knew that generic drugs were less costly. The reason for this lack of knowledge was understood to be the lack of proper dissemination of awareness about the drugs despite the prescribing practice of generic drugs in many areas [14]. Al-Gedadi et al. had observed that 71.7% of participants had not heard of generic medicines. In that study, 32% of the participants thought generic drugs to be more harmful [15]. This underlines the fact that awareness programs for the patients and the general public by the prescribers about any health or drug-related policy can increase the reach and acceptability of the same.

Lira et al., in their study, observed that only 48.6% of the participants knew about the entity called generic medicine. Their study found a noticeable gap in the knowledge of the participants about the prices and quality of the generic drugs [16]. Himmel et al. in their study found that one-third of the participants thought that generic drugs were of inferior quality because they were cheaper. The inference of associating the higher price with better quality in a drug product was observed more in participants above 60 years of age, who were chronically ill and were less educated [17]. In contrast to our study, Ahire et al. found that most of their patients (60.86%) were aware of the fact that generic medicines were comparable in efficacy to branded ones and that the marketing of generic drugs was also regulated by the same rules and regulations as the branded counterparts [18]. The patients in their study could have belonged to more educated strata as compared to our study which had patients who mostly visited the government-sponsored hospital and were mainly from a lower socioeconomic background. Das et al., in their study, found that 10% of patients considered generic drugs to be more harmful than branded ones, which was similar to as noted in our study [19].

In the national survey conducted by Shrank et al., only 37.6% agreed to use generic medicines for themselves. In their study, 53.7% of participants shared that their prescribers did not discuss the type of drugs being prescribed [20]. To fill the gaps in knowledge about generic medicines among patients and other stakeholders, the government should actively promote quality generic sales and also initiate awareness regarding the same. There should be campaigns and other promotional events regularly to educate people about the availability of generic medications and their quality, safety, and effectiveness. The government, non-governmental organizations, and leaders must use social media platforms such as Facebook, Instagram, Twitter, WhatsApp, etc. to educate the same among the masses. Other measures such as prescription audits should be undertaken at every hospital by competent authority to avoid overprescribing of expensive branded medications.

Limitations of the study

The study was conducted in a remote island state of the country to assess the outreach and awareness of the initiative, so it involved the data generated from patients visiting the only tertiary referral government hospital in the urban area of the islands with a single Jan Aushadhi outlet in the premises for the patients. The data on the topic may not be truly representative of the entire population in the Andaman and Nicobar group of islands with socioeconomic, cultural, and educational differences. Another limitation was the sample size of convenience with 200 participants. A larger sample size would have added a deeper insight into the findings.

## Conclusions

This is the first study regarding the awareness of the Jan Aushadhi program among the patients in these remote Indian islands. The study observed that the patients had very poor understanding of the Jan Aushadhi Scheme and generic medicines with the majority being ignorant and having incorrect information. The scheme launched by the government is a major step towards making quality medicines affordable and available to the common man. Still, despite the physicians prescribing through Jan Aushadhi outlets in the government setup or availability of the generic drugs dispensed at such centers, the knowledge about the scheme and the acceptability of the generics are far from satisfactory, and a more proactive approach by the healthcare workers and authorities is needed to disseminate the scheme-related facts and dispel the myths regarding generics to help the common man in accepting this unique, well-intended program wholeheartedly. 
